# The Cyclopædia of Practical Medicine

**Published:** 1836-01

**Authors:** 


ART. III.—*The Cyclopædia of Practical Medicine.* Edited by JOHN FORBES, M.D. F.R.S., ALEXANDER TWEEDIE, M.D., and JOHN CONOLLY, M.D.—Four vols. Royal 8vo. *London*, 1833, 1834, 1835.

IT will not be expected by our readers, considering the names which occupy the title-page of this review, that we should attempt to give any thing like a detailed analysis, much less a critical judgment, of the work of which we have now transcribed the title. Yet we feel that we should not be doing justice, either to our readers or the distinguished authors of the Treatises of the Cyclopædia, so recently concluded, if we omitted all notice of it. Independently of its merits as a separate work, its publication must be regarded as marking an important epoch in the medical [literary] history of this country. No attempt had been previously made in Great Britain to unite the great body of practical men of eminence, in any one branch of the profession, in a combined plan of embodying and giving to the world the actual state of our knowledge in that particular branch; and no individual had yet succeeded, though some had made the attempt, in producing such a work as might be fairly considered to represent the great and characteristic features of British medicine in these later times. The Cyclopædia has certainly done the one, and professes to do the other; and, although the relation in which we stand to it forbids us from giving any opinion as to the extent to which it has fulfilled its professions, nothing need preclude us from giving some account of the manner in which this has been attempted. This, however, we shall do very briefly, and in a way, we trust, not to compromise our own independence, or give offence to the most scrupulous of our readers.

The only works published in the English language previously to the Cyclopædia, and having any thing of a similar plan, are the four Dictionaries, by Drs. James, Motherby, Morris, and Parr. The first of these, comprised in three very large folio volumes, was published, the first in the year 1743, the second and third in the year 1745. This dictionary is on a most comprehensive plan, containing indeed all the collateral branches of medicine and surgery, as is evidenced by its title, "A Medical Dictionary; including Physic, Surgery, Anatomy, Chymistry, and Botany, in all their branches relative to medicine; together with a history of drugs, an account of their various preparations and uses." It is a work of very considerable merit, containing an infinity of important facts and details; and although consisting, in a great measure, of a continued series of extracts from preceding authors, put together very inartificially, and with only a scanty share of editorial cement, it must be allowed to be a convenient storehouse, where the reader may securely seek for, and will find, much that is recorded in the best works of preceding authors. The editor has, indeed, been singularly sparing of his comments; and, although he appears to have been so advisedly and on principle, we confess we should have gladly resigned a modicum of his borrowed opinions for some of his own. But Dr. James's modesty was equal to his learning. "If," says he, in his preface, "this work is a little more prolix than was at first proposed, the purchaser will find his account in it; and will, it is presumed, be more inclinable to pardon it, when he reflects upon the mortification an author must suffer whilst he sacrifices, to the interest of his readers, entire pages of his own productions; provided he can believe me to have a tenderness for my own literary offspring, equal to that of most other writers for their works; a supposition by no means unreasonable, especially when I assure him I have taken infinitely more pains to divest myself of prejudices in favour of any theory, system, or mode of practice whatever, than I have to conquer my affections." This preface, by the way, is a very extensive and learned history of physic, from the earliest times down to those immediately preceding his own, and well merits the reader's notice.

The compilation of Dr. Motherby was first published in the year 1776, in one volume folio. A second edition was published by the original editor, shortly after the first; and subsequent editions were given by Dr. Wallis, in 1791, 1795, and 1801; the work, in the last, being augmented to two volumes. This work, regarded as a Medical Dictionary, in the ordinary acceptance of that term, is superior to that of Dr. James: it contains much information of a more modern stamp, especially the later editions by Dr. Wallis; but, regarded in a medical point of view, or as a collection of monographs on medicine and the collateral sciences, it is greatly inferior, and is indeed scarcely of any use to the practitioner. It has few, if any, pretensions to be looked upon as a system of medicine; but it is by no means without value as a Medical Lexicon, and compendium of medical science at the time it was compiled. Its title is as follows: "A New Medical Dictionary, or General Repository of Physic; containing an explanation of the terms, and a description of the various particulars relating to Anatomy, Physiology, Physic, Surgery, Materia Medica, Chemistry, &c. &c."

The Dictionary usually known as that of Dr. Parr, although it is entitled "The London Medical Dictionary," is, in fact, only a greatly improved edition of the work of Dr. Motherby and Dr. Wallis. It is much better known than the preceding, and is indeed much more deserving of being known. We still think, however, that Dr. Parr made use much too largely of the work of his predecessors, to justify him in putting his work forth as an original. "The work," he says, in his preface, "is to be considered as original, and the names of Motherby and Wallis are consigned to the oblivion from which they had for a time escaped." This seems to us not merely to be spurning the dead lion, but to be doing so at the very time he is making use of the carcase to promote his own objects. Like its predecessors, the Dictionary of Dr. Parr comprised the whole of the medical and collateral sciences; and consequently, although its extent is considerable, (two thick volumes in quarto,) it could only give a meager epitome of any one department. The work fulfils the promise of its title as to variety of subjects, but it was impossible to give any thing like satisfactory views of any of them within such narrow limits. Every thing not borrowed from the works of Drs. Motherby and Wallis was, we believe, the production of Dr. Parr's own pen. The following is the title: "The London Medical Dictionary; including, under distinct heads, every branch of medicine, viz. Anatomy, Physiology, and Pathology, the Practice of Physic and Surgery, Therapeutics, and Materia

Medica; with whatever relates to Medicine in Natural Philosophy, Chemistry, and Natural History." It was published in the year 1809.

Two years before the publication of Dr. Parr's Dictionary,* a work, of the same general character, appeared at Edinburgh, under the avowed editorship of Dr. Robert Morris and Mr. Kendrick, surgeon, entitled "The Edinburgh Medical and Physical Dictionary; containing an Explanation of the Terms of Art in Anatomy, Physiology, Pathology, Therapeutics, Surgery, Midwifery, Pharmacy, Materia Medica, &c. &c.; also a copious account of Diseases, and their Treatment, &c." It was published in the same form, and is nearly of the same extent, as Dr. Parr's Dictionary, consisting, like it, of two thick volumes in quarto. This Dictionary is, on the whole, decidedly inferior to that of Dr. Parr. It is much more of a compilation, and indeed contains hardly any thing original, being avowedly composed, in a great measure, from the published works of preceding authors. Before the appearance of the London Dictionary, it was, however, the best modern Medical Dictionary in our language; and in some departments, especially the surgical, it is still superior to its more popular successor and rival: yet it never was, and never deserved to be, esteemed a work of good authority.

The Medical Dictionary of Quincy, afterwards so much improved by Dr. Hooper, and some other works of the same kind, do not require any notice in this place, as they were only intended as technological lexicons, and have no pretensions to be regarded as exhibiting a view of any of the departments of medical science.

On the continent of Europe alphabetical arrangements, or systems of medicine and the medical sciences, were undertaken on a scale of much greater splendour and extent, long before the publication of the Cyclopædia was thought of; and it is to them that we must look for the principles and plan on which the last-named work was constructed.

The first Medical Dictionary on a large scale published in France is that which constituted a part of the great *Encyclopédie Méthodique*, begun in the year 1782, and only very recently concluded. The portion of this great work devoted to the medical sciences (Dictionnaire de Médecine) amounts to fifteen volumes in quarto. Having commenced so far back as 1787, much of the information contained in the earlier volumes is obsolete, and the articles in the beginning of the alphabetical series form a remarkable contrast, in this respect, with those at its termination. It is written by a great many different authors, many of them of great eminence; and, on the whole, it is a work of great value, especially the latter volumes.

The next work of the same kind from the prolific press of France is the great *Dictionnaire des Sciences Médicales*: it commenced in the year 1812, and terminated in the year 1822, and consists of sixty volumes 8vo. As its title implies, it comprehends the whole of the medical sciences, and it numbered among its writers all the most eminent men of France. It is a work of unequal merit; but, taken as a whole, it comprehends more information than any other work of the kind that has been hitherto published. The very comprehensiveness of its

* We believe an edition of the London Dictionary of Medicine made its appearance in 1807 also, but was afterwards suppressed.

plan renders it unwieldy, and its great price limits its circulation. In some measure to remedy these inconveniences, an abridgment of it has been published in fifteen volumes 8vo., under the title *Dictionnaire abrégé des Sciences Médicales.* It commenced in 1821, and terminated in 1826.

Contemporaneously with this abridgment appeared the *Dictionnaire de Médecine*, the joint production of from twenty to thirty of the most distinguished men in the profession in France. Like its predecessors, it embraces all the branches of medicine. It terminated in 1828, and consists of twenty-one volumes 8vo. There is a new and much improved edition of this work now in course of publication, under the title *Dictionnaire de Médecine, ou Répertoire général des Sciences Médicales.* It contains new articles by the present editors, and considerable additions to the old, and is on the whole a very excellent work. It has been in the course of publication about three years, and has reached its twelfth volume.

This work was scarcely concluded when another, nearly on the same plan and extent, made its appearance, the *Dictionnaire de Médecine et de Chirurgie pratiques*, which is scarcely yet completed. It consists of fifteen volumes 8vo. As a practical work on medicine and surgery, this dictionary certainly excels all its predecessors: the articles are more select, and those on the most important subjects are much fuller than in the *Dictionnaire de Médecine*, and they are also on the whole better. It is the production of about twenty of the most celebrated physicians and surgeons of the present æra. This dictionary gives bibliographical notices at the end of the articles; a very laudable practice, which had not been adopted by its immediate predecessor. We ought to have mentioned that the bibliographical references in the *Dictionnaire des Sciences Médicales* are very extensive.

Several other nations on the European continent have also taken precedence of England in the publication of works of this kind, but none of them have exhibited the same activity as France. In Germany several have appeared, but only one, we believe, on precisely the same plan as the French dictionaries just noticed.

Of the smaller works, the *Encyklopädie der Heilwissenschaft*, by Burdach, in three vols. 8vo., is the best. The first volume of this was published as early as 1800, but a new edition of the whole was given in 1816.

A more extensive work is that published by Consbruch, Ebermaier, and Niemann, under the name of *Allgemeine Encyclopädie für praktische Aertzte und Wundärtzte*, in eleven volumes 8vo., commencing in 1815, and terminating in 1830. It is entirely written by the above-named authors, and consists of a series of manuals on the different departments of the Medical Sciences. It contains no original dissertations.

The *Encyclopädisches Wörterbuch der Medicinischen Wissenschaften*, now in the course of publication, is entirely on the same plan as the great French dictionaries, and partakes of all their excellencies and defects. It is edited by Graefe, Hufeland, Link, Busch, and Müller of Berlin, and enrols in its list of contributors the most distinguished writers of Germany. It was begun in 1828, and has now reached its twelfth volume, and the letter F.

Besides the above, there is now in course of publication, in Germany, a translation of the Dictionnaire de Médecine, by Meissner and Schmidt.

An abridged translation of the same work is also publishing *in Italian*, at Venice; it commenced in 1831: also a translation of the last French dictionary, and of the English Cyclopædia of Medicine; the former at Venice, the last at Leghorn.

Spain, although one of the last in the advance of medical science, can boast of more than one Medical Dictionary. In 1807, one was published at Madrid, by Ballano, in seven volumes 4to., to which a Supplement was added of four volumes more, by Hurtado, in 1823. Both these are mere compilations, chiefly from the French. Besides these there is a professed translation, or rather an abridgment, of the *Dictionnaire des Sciences Médicales*, which has already reached beyond the thirtieth volume, and is not yet complete.

The Cyclopædia of Practical Medicine coincides with the best of the continental dictionaries, in consisting of a series of original treatises written by a great number of different authors; but it differs from all of them in the greater comparative number of its writers, and in the more limited variety of its subjects. All the French Dictionaries, as we have seen, treat of the whole body of the medical sciences; the Cyclopædia, as its title indicates, contains only treatises on the nature and treatment of such diseases as come strictly within the domain of medicine, on materia medica, therapeutics, and medical jurisprudence; it takes no notice whatever of anatomy, physiology, surgery, chemistry, botany, &c., which occupy so large a portion of the Foreign Dictionaries. Notwithstanding this, the number of writers is comparatively much greater in the English work, being not fewer than sixty-seven, (and all physicians,) as we find by reckoning the names in the list prefixed to the first volume, while the names, as given on the title-pages of the Foreign Dictionaries, are, for the Dictionnaire des Sc. Méd., 81; the Dict. de Méd., 28; and the Dict. de Méd. et de Chir. Prat., 22. The number of writers in the great *Wörterbuch*, which promises to be almost as voluminous as the Dict. des Sc. Méd., although it was announced in the preface to consist only of twenty-five volumes, is seventy-three.

In consequence of the limitation of the work to the domain of Practical Medicine, the different subjects are, generally speaking, treated at much greater length than in the foreign works: and it might therefore be presumed that the distribution of the materials to so many writers must give a greater chance of originality, as well as comprehensiveness, than if every different writer, instead of limiting himself to one subject or class of subjects, had entered upon several. We are much mistaken if a comparison of the English with the foreign works will not prove this to be the case.

Prefixed to the first volume is a history of Medicine of considerable extent, written by Drs. Bostock and Alison; the former bringing it down to the commencement of the current century; the latter completing it to the present time. Appended to the whole is a very extensive Bibliography and a copious Index.

We will not permit ourselves to say anything more respecting this work in our character of critics, but shall conclude by laying before the reader a few extracts from the preface, yet further explanatory of its nature and objects, avoiding as much as possible all statements which involve mere opinions of the Editors, or which can in any way be judged to compromise our impartiality.

"The ambition of the Editors was not limited to the formation of a mere manual, fitted for those who only demand the smallest supply of exact intelligence with which practice can be carried on, or professional station maintained, without a palpable exposure of ignorance. Their object was no less than to prepare a compendium of the best parts of ancient and modern medicine, theoretical and practical; not passing over with disregard the vast literature of the ancient writers, but rather rescuing it from the voluminous oblivion in which much of it was lost; and also collecting with care the more accurate, condensed, and applicable knowledge of modern authors and of modern times into a liberal and consistent system, from works little known to the generality of English readers, and familiarly known to very few.

"Thus, whilst the great claims of the older cultivators of medicine have never been forgotten, the labours of the moderns, and more particularly of the French, German, and Italian pathologists, by which, conjointly with those of British practitioners, the whole face of practical medicine may be said to have been changed, have attracted the most diligent and thoughtful attention. The learned reader does not require to be assured that the task of reference for specific information to many older works, once of high and deserved authority, and still esteemed, is often both tedious and little profitable, whilst their ample volumes yet contain much valuable matter, not unworthy of preservation, and which it is no fruitless employment to endeavour to place along with the better arranged facts of later writers, in one view, before the practitioner and the student. Throughout the prosecution of this large design, it has never been forgotten that the Cyclopædia would be referred to by various readers for various objects: by the young practitioner as the guide and counsellor of practice, especially when beset with practical difficulties; by the older practitioner for complete and concise information, and for medical learning not scanty and illiberal, but without scholastic pedantry; and by the student for applicable knowledge, suited to the actual time, collateral with and auxiliary to his prescribed studies, and satisfactorily directing the efforts of his inquiring mind." (P. 2.)

"However natural and proper it may be for the Editors to take a retrospective survey of the work when it is brought to a conclusion, and to examine with what fidelity they have fulfilled their engagements, and how far and how equally the various departments of practical medicine have in their turn occupied their attention, it is hardly possible to express the results of this retrospection in words which will not expose them to the charge of being disposed to look back upon their exertions with too much complacency. Yet they may perhaps be allowed to say, that if the reader will take the trouble to inspect the mere titles of the articles contained in these columns, comprehending nearly three hundred original essays of known and distinguished authors, and will bear in mind either the leading physiological divisions of diseases, or consider them with reference to the head, the chest, the abdomen, the surface, or the general condition of the body; as well as the subjects of obstetrical medicine, materia medica, or medical jurisprudence; he will sufficiently appreciate the care bestowed to make the Cyclopædia satisfactory to all who refer to its pages, and at the same time strictly a book of practical reference. No subject, it is believed, immediately practical in its nature or application, has been left out, although unnecessary disquisition has been as much as possible avoided.

"It has consistently entered into their plan to admit of a far wider range of subjects than appears heretofore to have been considered necessary in works professedly written on the practice of medicine, but a range comprising many new subjects of extreme importance to those engaged in practice or preparing for it. Such are the subjects of Abstinence, Acupuncture, Age, Change of Air, Antiphlogistic Regimen, Asphyxia, Auscultation, Bathing, Bloodletting, Morbid States of the Blood, Climate, Cold, Contagion, Convalescence, Counter-irritation, Derivation, Congestion and Determination of Blood, Dietetics, Disinfection, Physical Education, Electricity, Endemic Diseases, Epidemics, Expectoration, Exploration of the Chest and Abdomen,

Galvanism, Hereditary Transmission of Disease, Induration, Irritation, Infection, Latent Diseases, Malaria and Miasma, Perforation, Prognosis, Pseudo-morbid Appearances, Pulse, Softening, Medical Statistics, Stethoscope, Sudden Death, Symptomatology, Temperament, Toxicology, Transformations, Transfusion, Tubercle, Ventilation, Mineral Waters; and those of various general articles on the pathology of organs." (P. 4.)

"The Editors will only add, that they have avoided multiplied and artificial divisions, and have aimed at that plainness of arrangement which most facilitates a ready and immediate reference. They have also shunned the fault of encumbering medical literature with new and uncouth terms, always preferring those in common use, and of which the signification was the least doubtful. It has been their constant desire to guard the most inexperienced reader from distraction in the pursuit of practical knowledge, and to assist the more advanced reader in the grouping and generalization of the ideas with which his personal experience may have imbued him." (P. 8.)

